# Characteristics of inmates witnessing overdose events in prison: implications for prevention in the correctional setting

**DOI:** 10.1186/1477-7517-6-15

**Published:** 2009-07-09

**Authors:** Carmen E Albizu-García, Adriana Hernández-Viver, Jacqueline Feal, José F Rodríguez-Orengo

**Affiliations:** 1 Center for Evaluation and Sociomedical Research, University of Puerto Rico Medical Sciences Campus, San Juan, Puerto Rico; 2 Clinical Psychology Program, Carlos Albizu University, San Juan, Puerto Rico; 3 Institute for Forensic Medicine, San Juan, Puerto Rico; 4 Current address: Department of Biochemistry, University of Puerto Rico Medical Sciences Campus, San Juan, Puerto Rico

## Abstract

**Background:**

Although prevention of opiate overdose has been gaining attention as a harm reduction measure with community drug users, there is scarce information about drug overdose in prison. In correctional institutions without a drug free environment, awareness of overdose events is an important public health concern. This study explores the frequency with which inmates in a state penitentiary system report having witnessed drug overdose events in prison. It also explores whether participants who have witnessed an overdose in prison and know someone who died from an overdose in prison significantly differ from those that do not in selected sociodemographic variables and drug use history to identify a target population for prevention interventions.

**Methods:**

Data comes from a cross-sectional survey of sentenced inmates in the state prisons of Puerto Rico. A complex probabilistic, multistage sampling design was used. A total of 1,179 individuals participated for an 89% response rate.

**Results:**

Factors associated with witnessing an overdose event in prison include: male sex, age 25 or older, drug use during current incarceration, and drug injection in prison. Factors associated with knowing someone who died from an overdose in prison include: male sex, age between 25–35, previous incarcerations, and drug use during current incarceration.

**Conclusion:**

Witnessing a drug overdose is a frequent occurrence within the prison system. The likelihood of witnessing an overdose is greater with being male, polydrug use and drug injection in prison. Findings signal an urgent public health challenge that requires prompt interventions to reduce this drug related harm within the correctional system, including adequate access to medication with opiate agonists.

## Background

Although prevention of opiate overdose has been gaining attention as a harm reduction measure with community drug users [1-3], there is scarce information regarding the frequency with which drug users experience an overdose while in prison. Prison inmates use diverse illicit drugs during incarceration even if it is presupposed that drugs are unavailable in correctional institutions [4-6]. The literature addressing drug overdose events in criminal justice populations has focused on recently released ex-inmates who are at increased risk of both fatal and nonfatal overdose events compared to community drug users, [7-11], likely as a result of a loss of tolerance during confinement [12,13] and/or to polydrug use, particularly the concurrent use of Central Nervous System (CNS) depressants, such as alcohol and benzodiazepines [14].

While the United States (U.S.) has the largest prison population worldwide [15], a large proportion of which has used an illegal drug within the 30 days prior to arrest [16,17], there is a lack of research addressing the occurrence of opiate overdose events in the US prison system. In spite of the political difficulties associated with recognizing that illicit drug use occurs in prisons, overdose is a frequent event among community drug users and a potential complication of illegal drug use during incarceration. A study that interviewed drug users in New York City found that nearly 58% had witnessed at least one heroin overdose within the preceding three years [18]. From a public health point of view, the prevention of premature death, whether due to accidental overdose or suicide, is of paramount importance [19]. Complications from drug overdose include serious clinical conditions such as pulmonary edema, cardiac arrhythmia, cognitive impairment, rhabdomyolysis, and indirect physical injury [14,20-22].

In correctional institutions that do not have a drug free environment, awareness of the possible occurrence of fatal or non fatal overdose events is an important public health concern. Since most overdose events occur in the presence of witnesses [23,24], this study explores the frequency with which inmates report having witnessed drug overdose events while in the prison setting. We explore whether inmates reporting to have witnessed an overdose event or overdose death in prison, significantly differ from those that do not in selected sociodemographic variables and drug use history. This information is essential to identify a target population for prevention interventions. We also explore respondents' willingness to assist a fellow inmate that appears to be experiencing a drug overdose. Training heroin users to respond to overdose events among their peers is a public health intervention designed to reduce fatalities that is increasingly included in comprehensive public health responses [25].

## Methods

This study uses data obtained from a cross-sectional survey of sentenced inmates in the state prisons of Puerto Rico commissioned by the Puerto Rico Department of Correction and Rehabilitation in 2005 to assess drug treatment needs and inform health services planning [26]. The sample for this study consisted of 1,331 randomly selected sentenced inmates from 26 penal institutions, out of 39 existent in the Puerto Rico prison system during 2004, representing 13% of the total sentenced inmate population [Department of Corrections and Rehabilitation, personal communication, 2005].

A complex probabilistic, multistage sampling design was developed based on four sampling stages. The first stage consisted of stratifying by type of institution: adult men, juvenile men, and women. For the second sampling stage, institutions were stratified based on the prevalence of positive urine tests for illegal drugs as reported by the prison authorities. Using as reference the prevalence of illicit drug use in the general population of Puerto Rico, institutions were grouped in four categories (prevalence "normal" or comparable to that of the general population, 1%–14%; high, 15%–25%; very high, 26%–50%; and unknown). Two studies provided estimates of the prevalence of drug use in the general population of Puerto Rico. Canino and colleagues (1993) reported a prevalence of illicit drug use of 8.2% and, eight years later, Colón and colleagues (2000) estimated a prevalence of 10.7% [27]. For the third stage, institutions were furthered stratified by security level (Maximum, Medium, Minimum, and Admission Center). If there were more than two institutions at a given security level, two were randomly selected, with probability of selection proportional to the institutions' population. Finally, for each of the 26 institutions selected and depending on their size, a random sample was obtained comprising 5% to 39% of the inmate population. Subject selection was carried out anonymously by the research team two days prior to initiating interviews. A total of 1,179 individuals participated in the study for an 89% response rate.

Two computerized interview modalities were used for data gathering: CAPI (Computer Assisted Personal Interview) and ACASI (Audio Computer Assisted Self Interview). Trained interviewers conducted the computerized personal interview and were available to answer questions during the self-administered interview. The study was reviewed and approved by the University of Puerto Rico Medical Sciences Campus Institutional Review Board.

### Measures

The Computer Assisted Personal Interview modality (CAPI) was used for questions assessing sociodemographic characteristics, including gender, age at interview, education, previous incarcerations, and health status. Participants were also asked if they had ever experienced an overdose event. The Audio Computer Assisted Self Interview (ACASI) was used for questions regarding illicit drug use, behaviors that constitute risk for contagion with blood borne pathogens, frequency of drug use in prison, and route of administration. In addition, participants were asked if *they have observed in prison someone experiencing an overdose a*nd whether *they know of someone who died of an overdose in prison*. Subsequently they were asked about their disposition to offer assistance to someone experiencing an overdose in prison.

### Statistical Analysis

The Questionnaire Development System (QDS) version 2.1 was used to program both the CAPI and ACASI questionnaires. Data was transported and analyzed using the Statistical Analysis System (SAS version 9.1). The chi-square test was used to test the association between sociodemographic and drug use characteristics with witnessing an overdose in prison as well as for knowing someone who died from an overdose in prison. Multivariate logistic regression was used to determine sociodemographic and drug use characteristics associated with witnessing an overdose in prison and knowing someone who died from an overdose in prison. For the logistic regression, all variables were entered at the same time. Significance level for all analysis was established at p < 0.05.

## Results

### Descriptive data and bivariate analysis

Table 1 summarizes descriptive data for the total sample as well as the distribution of sample characteristics by witnessing an overdose event and knowing someone who died from an overdose in prison, our two dependent variables.

**Table 1:** Sociodemographic and drug use characteristics by witnessing or knowing someone died from overdose in prison

	**Total Sample** **N = 1,179**	**Witnessed someone** **suffering an overdose** **in prison (n = 1,155)**^a^	**p value**	**Know someone** **dying from an** **overdose in prison** **(n = 1,157)**^b^	**p value**
		**Yes (n = 525, 45.5%)**		**Yes (n = 390, 33.7%)**	
	**n (%)**	**n (%)**		**n (%)**	
**Gender**					
Male	959 (81.3)	488 (52.1)	p < 0.001	361 (38.5)	p < 0.001
Female	220 (18.7)	37 (17.0)		29 (13.3)	
**Age**					
18–24 years	277 (23.5)	84 (31.0)	p < 0.001	72 (26.6)	p = 0.001
25–35 years	596 (50.5)	305 (52.4)		225 (38.7)	
36 or more	306 (26.0)	136 (45.0)		93 (30.6)	
**Education level**					
9^th ^grade or less	449 (38.1)	175 (39.7)	p = 0.006	145 (32.9)	p = 0.726
10–12^th ^grade	561 (47.6)	274 (49.7)		192 (34.8)	
More than 12^th ^grade	169 (14.3)	76 (46.6)		53 (32.1)	
**Previous incarcerations**					
First time	427 (36.3)	158 (37.8)	p = 0.001	98 (23.4)	p < 0.001
1–5 times	603 (51.2)	291 (49.1)		236 (39.8)	
More than 5 times	147 (12.5)	75 (52.4)		55 (38.2)	
**Institution's prevalence of positive urine tests**					
Very high	206 (17.5)	119 (58.9)	p < 0.001	86 (42.4)	p = 0.013
High	320 (27.1)	150 (47.9)		102 (32.7)	
Normal	399 (33.8)	158 (40.2)		133 (33.8)	
Unknown	254 (21.6)	98 (39.7)		69 (27.8)	
**Experienced an overdose (lifetime)**					
Yes	144 (12.2)	86 (60.1)	p = 0.001	63 (44.1)	p = 0.005
No	1035 (87.8)	439 (43.4)		327 (32.3)	
**Illicit drug use (lifetime)**					
Yes	1033 (87.6)	483 (47.5)	p = 0.001	361 (35.5)	p = 0.001
No	146 (12.4)	42 (30.4)		29 (20.7)	
**Drug injection before entering prison**					
Yes	323 (27.5)	194 (60.6)	p < 0.001	144 (44.9)	p < 0.001
No	852 (72.5)	330 (39.6)		245 (29.4)	
**Drug and alcohol use during this incarceration^**					
None	632 (53.6)	349 (36.9)	p < 0.001	115 (18.6)	p < 0.001
Only one type of drug/alcohol	160 (13.6)	25 (89.3)		62 (39.5)	
Polydrug with/without alcohol	387 (32.8)	148 (83.1)		213 (55.8)	
Drug injection in prison*					
None	966 (82.3)	349 (36.9)	p < 0.001	262 (27.6)	p < 0.001
Drug injection, no risky behaviors	29 (2.5)	25 (89.3)		17 (60.7)	
Drug injection, risky behaviors	178 (15.2)	148 (83.1)		110 (61.8)	

a, b Although sample size was 1,179, due to missing values, only participants who responded to these items were included in the analysis.

^ Drugs included are marihuana, crack, cocaine, heroin, speedball and/or tranquilizers.

*Risky behaviors include sharing needle equipment, not cleaning needle equipment before injecting, reusing water, backloading, doesn't clean skin prior injecting, sharing cooker and cotton and reusing needles

Although sample size was 1,179, only 1,155 participants responded to the question assessing witnessing an overdose event in prison, while 1,157 responded to the question assessing if they knew someone who died from an overdose while in prison. Results show that 45.5% of the population witnessed an overdose in prison while 33.7% knew someone who died from an overdose in prison. Bivariate analyses were conducted to test associations between the independent variables and each of the dependent variables. Significant associations were found for being male (witnessing overdose reported by 52.1% of males vs 17.0% of females; knowing someone who died from an overdose in prison was reported by 38.5% of males vs. 13.3% of females), and age between 25 and 35 years (52.4% witnessed an overdose while 38.7% knew someone who died from an overdose in prison). Educational level proved significant with witnessing an overdose in prison: 49.7% of participants between the 10^th ^and 12^th ^grade reported witnessing an event when compared to other educational levels. Associations were also found for previous incarcerations: 52.4% of those with more than five prior incarcerations had witnessed an overdose event in prison and 39.8% knew of an overdose death.

Among the drug use variables, lifetime use of illicit drugs was significantly associated with witnessing an overdose (47.5%) and with knowing someone who died from an overdose in prison (35.5%). Significant associations were also found for drug injection before entering prison, with 60.6% having witnessed an overdose and 44.9% knowing someone who died from an overdose in prison. A larger proportion of inmates from institutions with very high prevalence of positive urine toxicology had witnessed an overdose (58.9%) or knew someone who died from an overdose in prison (42.4%). Among inmates reporting ever experiencing an overdose, 60.1% had witnessed an overdose and 44.1% knew of an overdose death in prison.

Significant associations were also found for variables related to drug use while in prison. Those who used only one type of drug or alcohol in prison (89.3%) had witnessed an overdose event, whereas polydrug use during confinement was significantly associated with knowing someone who died from an overdose in prison (55.8%),

Injecting drugs while in prison was significantly associated with witnessing an overdose event and knowing of an overdose death. Nearly two-thirds (61.8%) of participants who injected drugs in prison and incurred in behaviors that increase risk for contagion with blood-borne pathogens (such as sharing and/or not cleaning injection equipment, reusing water, backloading, not cleaning skin prior to injecting, sharing cooker and cotton, and reusing needles) knew someone who died from an overdose in prison.

### Multivariate analysis

Table 2 presents results obtained from the multivariate logistic regression analysis performed to explore the adjusted effects of the independent variables on each of the two dependent variables.

**Table 2:** Logistic regression of factors associated with witnessing or knowing someone died from overdose in prison

	**Witnessed someone suffering an overdose in prison (n = 1,153)^a^**		**Know someone dying from an overdose in prison (n = 1,155)^b^**	
	AOR** (95% CI)	**p value**	AOR** (95% CI)	**p value**
**Gender**				
Female	1.0	-	1.0	-
Male	6.2 (3.9–9.8)	p < 0.001	4.5 (2.8–7.2)	p < 0.001
**Age**				
18–24 years	1.0	-	1.0	-
25–35 years	2.4 (1.7–3.4)	p < 0.001	1.5 (1.0–2.1)	p = 0.032
36 or more	2.5 (1.6–3.8)	p < 0.001	1.3 (0.8–1.9)	p = 0.304
**Education level**				
9^th ^grade or less	0.7 (0.5–1.2)	p = 0.199	1.0 (0.7–1.6)	p = 0.953
10–12^th ^grade	1.0 (0.7–1.6)	p = 0.886	1.0 (0.6–1.5)	p = 0.973
More than 12^th ^grade	1.0	-	1.0	-
**Previous incarcerations**				
First time	1.0	-	1.0	-
1–5 times	1.1 (0.8–1.5)	p = 0.556	1.7 (1.3–2.4)	p = 0.001
More than 5 times	1.6 (1.0–2.7)	p = 0.054	1.8 (1.1–2.9)	p = 0.014
**Institution's prevalence of positive urine tests**				
Very high	0.9 (0.6–1.4)	p = 0.681	0.7 (0.4–1.0)	p = 0.058
High	0.9 (0.6–1.2)	p = 0.398	0.6 (0.4–0.9)	p = 0.005
Normal	1.0	-	1.0	-
Unknown	0.7 (0.5–1.1)	p = 0.151	0.5 (0.4–0.8)	p = 0.003
**Experienced an overdose (lifetime)**	1.4 (0.9–2.3)	p = 0.172	1.2 (0.8–1.8)	p = 0.412
**Illicit drug use (lifetime)**	0.7 (0.5–1.2)	p = 0.191	0.8 (0.5–1.3)	p = 0.369
**Drug injection before entering prison**	1.1 (0.8–1.7)	p = 0.514	1.1 (0.7–1.6)	p = 0.663
**Drug and alcohol use during this incarceration^**				
None	1.0	-	1.0	-
Only one type of drug or alcohol	3.1 (2.1–4.7)	p < 0.001	2.9 (1.9–4.4)	p < 0.001
Polydrug with/without alcohol	7.1 (4.8–10.4)	p < 0.001	4.2 (2.9–6.1)	p < 0.001
Drug injection in prison*				
None	1.0	-	1.0	-
Drug injection, no risky behaviors	5.9 (1.4–24.5)	p = 0.014	1.5 (0.6–3.6)	p = 0.353
Drug injection, risky behaviors	1.9 (1.1–3.3)	p = 0.022	1.4 (0.9–2.2)	p = 0.177

a, b Although sample size was 1,179, due to missing values, only participants who responded to these items were included in the analysis.

^ Drugs included are marihuana, crack, cocaine, heroin, speedball and/or tranquilizers.

*Risky behaviors include sharing needle equipment, not cleaning needle equipment before injecting, reusing water, backloading, doesn't clean skin prior injecting, sharing cooker and cotton and reusing needles.

** Adjusted Odds Ratio

Results show that being male, polydrug use and drug injection in prison were strongly associated with witnessing an overdose, while being male and polydrug use were strongly associated with knowing someone who died from an overdose in prison. The odds of male inmates witnessing someone suffering an overdose in prison was more than six times that of female inmates (OR = 6.2; 95% CI = 3.9–9.8) while the odds of male inmates knowing someone who died from an overdose in prison was four and a half times higher than that of female inmates (OR = 4.5; 95% CI = 2.8–7.2). Regarding drug use during this incarceration, participants who are polydrug users had odds seven times higher (OR = 7.1, 95% CI = 4.8–10.4) of witnessing an overdose in prison compared to those who do not use alcohol and drugs in this incarceration. Moreover, the odds of polydrug users knowing someone who died from an overdose in prison were four times higher (OR = 4.2, 95% CI = 2.9–6.1) compared to participants who do not use alcohol and drugs during this incarceration. When analyzing drug injection in prison, results show that participants who inject in prison but do not incur in risky behaviors had odds almost six times higher (OR = 5.9, 95% CI = 1.4–24.5) of witnessing an overdose compared to participants that do not inject in prison. For participants who inject in prison and incur in risky behaviors, the odds were almost two times higher (OR = 1.9, 95% CI = 1.1–3.3) of witnessing an overdose in prison compared to participants that do not inject in prison. Although results show that odds are higher for drug injection in prison with no risky behaviors than for those who do incur in risky behaviors, this could be due to the fact that only 29 participants indicated drug injection with no risky behaviors. The majority of participants who do inject in prison incur in risky behaviors.

Additional results show that participants whose age is between 25 and 35 years had odds of witnessing an overdose in prison two times higher (OR = 2.4; 95% CI = 1.7–3.4) compared to participants between 18 and 24 years of age. The odds of participants between 25 and 35 years knowing someone who died from an overdose in prison were one and a half times higher (OR = 1.5, 95% CI = 1.0–2.1) compared to participants between 18 and 24 years. Participants whose age was 36 years or more had odds of witnessing an overdose in prison two and a half times higher (OR = 2.5, 95% CI = 1.6–3.8) compared to those between 18 and 24 years. Number of previous incarcerations was associated with knowing someone who died from an overdose in prison. Participants who have been incarcerated more than five times (excluding this incarceration) had odds almost two times higher (OR = 1.8; 95% CI = 1.1–29.9) of knowing someone who died from an overdose in prison compared to those incarcerated for the first time.

Participants from institution's with a high prevalence of positive urine tests had odds almost two times higher (OR = 1.7, 95% CI = 1.1–2.5) of knowing someone who died from an overdose in prison compared to those institutions whose prevalence is normal. Also, institution's whose prevalence of positive urine tests is unknown had an odds ratio two times higher (OR = 2.0, 95% CI = 1.3–2.5) of knowing someone who died from an overdose compared to those institutions whose prevalence is normal.

## Discussion

The present study used an existing data set from a survey assessing inmates' drug treatment needs that included questions related to overdose events as part of a large array of items exploring adverse outcomes associated with drug use in a large prison system. The results of this exploratory study indicate that witnessing a drug overdose or knowing someone who died from one may be a frequent occurrence within this particular context, highlighting the need for attention to these preventable events in this prison system. Although the prevalence of non-fatal overdose among opiate users varies among cities and age groups [13], the finding that nearly half of male inmates in this study belief they have witnessed a drug overdose during the current or a prior incarceration and nearly a third report that they know someone who died from an overdose in prison, suggests that this event is occurring in proportions comparable to what is reported from studies conducted with community drug users in other countries. Studies conducted in the U.S. and Australia show that over 50%–70% of injection drug users have witnessed a fatal or non-fatal opiate overdose during their lifetime [18,28-30]. The absence, to the best of our knowledge, of published reports estimating the prevalence of witnessing overdose events by inmates in US prisons and jails precludes assessing the extent to which these findings are idiosyncratic to this particular prison context. We also lack published information from surveys conducted with community drug users in Puerto Rico to compare the frequency with which they have witnessed an overdose event to that of our study population.

It is possible that in Puerto Rico, which is designated as a High Intensity Drug Traffic Area (HIDTA) since 1994 [31], there may be readier access to heroin in prison to account for the proportion of participants that report witnessing overdose events in this context. In response to survey questions that explored drug users perceptions of the ease with which drugs were obtained in prison compared to the free community, nearly 41.9% of inmates reporting use of heroin and/or speedball during confinement, that participated in the primary study, indicated that it took them less or approximately the same time to access drugs within the penal system (data not shown).

Multivariate analysis conducted for each of the dependent variables, witnessing an overdose event and knowing someone who died from an overdose in prison, generates models in which the likelihood of witnessing an overdose event increases with age, with being male, and with illicit drug use in prison, the odds ratio being greater if the respondent admits to polydrug use. The effect of a history of previous incarcerations is significant only for knowing someone who died from an overdose in prison. Collinearity between age and incarceration history likely explains the difference between the two models.

These findings signal an urgent public health challenge that requires prompt interventions to reduce this drug related harm within the correctional system and warrant assessing the feasibility and the effectiveness of peer interventions among drug users in prison. Results showed that 83.3% of study participants would be willing to learn how to intervene if they encounter an overdose event in prison (data not shown). Even though it is possible that the interview elicited a socially desirable response, given the very large proportion of participants that responded affirmatively, there is evidence from other studies that drug users are willing to provide resuscitative measures if adequately trained [23,32]. Drug using prison inmates may be a valuable resource for reducing overdose fatalities through training and the dissemination of new drug technologies such as naloxone. Further studies that clarify structural factors and staff attitudes that facilitate or hinder the implementation of overdose prevention programs in prison are required. In addition, there needs to be adequate access to medication with opiate agonists such as methadone and buprenorphine. Trends in overdose deaths in British Columbia suggest that expanded access to methadone treatment likely explains reductions in deaths observed across time [33]. Although with limited capacity, the PR Department of Correction and Rehabilitation has established, since 2002, opiate agonist treatment, initially with methadone and recently adding Buprenorphine-naloxone. Both modalities have been found acceptable to inmates and to the treatment sector in this particular correctional setting [34,35] and efforts are underway to increase the number of treatment slots to address the problematic use of opiates as well as to reduce recidivism during community re-entry.

In spite of the effectiveness of treatment in reducing adverse events associated with heroin use [36,37], treatment may not suffice. Darke and colleagues (2005) found that the risk of overdose diminished the longer participants stayed in treatment, but that a greater number of separate treatment episodes lead to an increase in overdose risk [38]. These findings imply that to address the challenge of overdose events within prisons, drug treatment programs' policies regarding inclusion and retention in treatment need to be less restrictive to avoid unwarranted treatment termination and focus on the participants' needs. Strict inclusion rules may increase the risk of discharge, with a subsequent increase on the mortality rate [39,40]. A comprehensive response will also require additional harm reduction measures. In a study with community drug users in Australia, a recent overdose experience was not a motivating factor for treatment initiation [41]. France and Spain, which adopted a series of harm reduction policies, have seen a reduction in drug overdose [42].

Several limitations need to be considered when interpreting these findings. The present study did not provide an operational definition of overdose to maximize the reliability of responses. It is possible that individuals with drug use experience were more likely than those naive to drug use to have identified an event as an overdose, which would overestimate the effect that being a drug user has on the likelihood of witnessing this event. In spite of this limitation, it is precisely in the network of drug users that overdose events are more likely to occur and the characteristics of drug use shared by this population sub-group need to be taken into account in designing prevention interventions. In addition, we cannot assess from this study the last year or lifetime prevalence of a fatal or non-fatal drug overdose in prison, since we are unable to determine the frequency with which the same event is reported by more than one participant. In the study sample, nearly two-thirds of the population had been previously incarcerated. The study does not pretend to report prevalence but to explore if drug overdose poses a significant health risk to imprisoned drug users and raise awareness of the need to improve our understanding and develop appropriate responses to this event in the correctional setting. Nevertheless, triangulation with data from the Puerto Rico Institute of Forensic Sciences for the period of 2002 through 2007 indicates that overdose deaths are indeed occurring within this prison setting. Of 351 deceased prison inmates transferred from the prison system to their premises for an autopsy report during this period, 36% were due to intoxication with an illicit drug. In 2004, the year prior to this study, 50 cases were reported with 19 related to drug use. Most of the drug-related deaths were men (95%) with an average age of 34.7 years (range 25–52) (Rodriguez-Orengo, J., personal communication, 2009).

## Conclusion

Witnessing a drug overdose is a frequent occurrence within the prison system in which the study took place. The likelihood of witnessing an overdose event increases with age, with being male, and with illicit drug use in prison, the odds ratio being greater if the respondent admits to polydrug use. These findings signal an urgent public health challenge that requires prompt interventions to reduce this drug related harm within the correctional system and warrant assessing the feasibility and the effectiveness of peer interventions among drug users in prison. In spite of the difficulties in accepting drug use in prison, administrators of correctional facilities need to assess the extent to which this preventable event is occurring. Adequate access to medication with opiate agonists needs to be part of a comprehensive public health response.

## Competing interests

The authors declare that they have no competing interests.

## Authors' contributions

CAG designed the final version of the study, wrote the discussion section and part of the introduction and conclusion, revised the abstract, methods and results section, and conducted additional literature search. AHV wrote the method and results section, performed all data analysis, prepared the tables, wrote part of the introduction and conclusions, wrote part of the abstract, and conducted additional literature search. JF designed the first version of the study, conducted literature review, and wrote a preliminary draft. JFRO wrote part of the discussion section. All of the authors contributed to and approved the final version of the manuscript.
